# Standardized Follow-Up Recommendations Improve Reporting of Incidental Renal Lesions in a Community Setting

**DOI:** 10.7759/cureus.40828

**Published:** 2023-06-22

**Authors:** Michael G Johnston, Skyler Burke, Chance M Brock, Saralyn Beckius, Scott King

**Affiliations:** 1 Medicine, Washington State University, Spokane, USA; 2 Radiology, Providence Sacred Heart Medical Center, Spokane, USA; 3 Radiology, Inland Imaging, Spokane, USA; 4 Radiology, Washington State University, Spokane, USA

**Keywords:** health care quality assurance, quality improvement tool, follow-up algorithm, follow-up parameters, incidental renal mass

## Abstract

Introduction

The objective of this quality improvement study was to assess radiology report follow-up recommendation trends upon detection of incidental renal lesions before and after instituting standardized follow-up macros.

Materials and methods

A retrospective review was performed in 2019 of multiphase imaging workups on renal lesions (n = 396), including the following imaging modalities: ultrasound, CT with and without contrast, and spine MRI. Utilizing the same collection methods, a similar retrospective set of cases was collected in 2021, 12 months following the creation of the renal follow-up macros (n = 501). After exclusions, the second set was left with 98 cases of newly characterized incidental renal lesions. For both sets, we assessed the reports of the exams that initially detected the incidental renal lesion. We evaluated the incident reports for the presence of a follow-up recommendation, recommendation completeness, and alignment with the American College of Radiology (ACR) white paper suggestions for renal lesion follow-up.

Results

Before the implementation of the standardized renal follow-up macros, initial follow-up recommendations were in concordance with the ACR white paper recommendations in 33 of 98 cases (33.7%), incomplete or discordant in 49 of 98 (50.0%), and absent in 16 of 98 cases (16.3%). Following the institution of our macros, there was an improvement in concordant follow-up recommendations (51/98; 52.0%) (p = 0.009), a decrease in the number of incomplete or discordant recommendations (37/98; 37.8%), and a decrease in the number of reports lacking a follow-up recommendation (10/98; 10.2%).

Conclusion

Utilization of standard language renal lesion follow-up macros improves the rate of appropriate follow-up recommendations in radiology reports when encountering a previously unknown incidental renal lesion.

## Introduction

Renal cell carcinoma (RCC) is the eighth most common cancer, representing 4.1% of all new cancer diagnoses in the U.S. as of 2019 [1]. In the absence of clinical signs such as hematuria, flank pain, or palpable flank mass, imaging remains the most common means of detecting RCC. Renal cancer accounted for more than 13,000 fatalities in the United States in 2021, making it the 11th most common cause of oncologic mortality [2]. More than 50% of all RCC is now diagnosed in asymptomatic patients as an incidental imaging finding [3]. O’Connor et al. demonstrated that up to 14% of patients undergoing non-contrast CT demonstrated indeterminate renal lesions measuring >1 cm even after excluding macroscopic fat-containing lesions, simple-appearing cysts, and hyperdense cysts [4].

Compounding the problem is low adherence by radiologists to follow-up imaging recommendations for incidental findings [4], including incidentally detected renal lesions. Additionally, variable concordance between radiology reports and societal guidelines is evident and documented throughout current practice [5]. This discordance of radiology follow-up recommendations from national and consensus guidelines has been demonstrated for a variety of incidental imaging findings in various organ systems. For example, a national survey conducted by Eisenberg et al. found radiologist concordance with Fleischner guidelines in only 24.7-60.8% of reports [6]. Another study of radiologists describing pancreatic cysts demonstrated the inclusion of a follow-up imaging recommendation in only 17.3% of reports [7]. To add further evidence, a similar study by Hanna et al. found that in an ER setting, patients with incidental findings of any kind on CT demonstrated discordance with societal recommendations in 29.8% of reports [8].

The frequency of incidentally detected renal lesions, both benign and malignant, underscores the need for appropriate, efficient workup and/or follow-up of incidental renal lesions. In most instances, the imaging workup of newly detected indeterminate incidental renal lesions lends itself to standardized follow-up algorithms. In the radiology literature, the most widely known follow-up algorithm for incidental renal lesions can be found in the ACR’s 'Management of the incidental renal mass on CT: A white paper of the ACR Incidental Findings Committee' [9]. Modern radiology dictation systems can accommodate personalized or systematic deployment of standardized dictation macros that are well-equipped to address most encountered scenarios. Through a thorough review of the current literature, it is shown that there is minimal data examining the use of standardized dictation macros pertaining to renal lesions, leading to this novel study with little precedence set.

In 2020, our large private-practice radiology group deployed a series of novel standardized follow-up macros for incidentally discovered renal lesions as part of a quality-improvement project. The objective of this project was to assess the impact of these macros on radiology report recommendations when encountering a new, asymptomatic renal lesion.

## Materials and methods

Pre-intervention baseline

We retrospectively gathered baseline radiology report data between January 2019 through December 2019, which we will refer to as “pre-intervention” data. Reports from contrast-enhanced abdominal MRI and multiphase abdominal CT scans were collected if they were performed for the workup of renal lesions (n = 298). Report indications were evaluated for the terms “renal” or “kidney” and “mass” or “lesion” or “cyst.” We then assessed the imaging report preceding the multiphase renal MRI or CT, to identify the imaging exam on which the lesion was initially detected, which we refer to as the “incident exam.” The provided clinical data from the incident exam was reviewed to determine if the lesion was incidentally detected. We excluded cases in which patients were symptomatic of a possible renal mass (hematuria, flank pain, palpable flank mass), or had a known history of pre-existing renal lesions. We excluded CT urograms, as the majority of these were performed on symptomatic patients. Other exclusion criteria included: patients under 18 years old, patients with Von Hippel Lindau or tuberous sclerosis, patients with multi-organ metastatic disease, patients with infiltrative renal or urothelial masses, cases lacking the initial radiology report, and cases in which no renal mass was present (Table 1). After exclusions, there were 98 patient reports with newly detected incidental renal lesions. 

**Table 1:** Criteria for inclusion or exclusion of data

Study Characteristics	Inclusion Criteria	Exclusion Criteria
Report Indication	Renal, Kidney, Mass, Lesion, Cyst	Anything not in included list
Sex, male-female ratio	Multiphase renal MRI and CT, Renal US	CT Urogram
Associated Symptoms	Anything not in excluded list	Hematuria, flank pain, palpable flank mass
Existing Diagnosis	Anything not in excluded list	Known existence of renal lesion, multi-organ metastatic disease, infiltrative renal or urothelial mass.
Other Criteria	Anything not in excluded list	No initial radiology report, no renal mass present

The 98 incident exam reports were evaluated to assess the appropriateness of initial radiology recommendations. We utilized the ACR white paper as the standard for determining recommendation appropriateness [9]. The reports were divided into three categories of report recommendation, including: 1) no report recommendation offered, 2) incomplete or discordant recommendation, or 3) correct recommendation. Reports were categorized as incomplete if they lacked details such as not recommending an imaging modality or recommended timeframe for imaging follow-up, when appropriate. Recommendations that deviated from the ACR white paper recommendations were categorized as discordant. Recommendations judged to be concordant with ACR white paper recommendations were considered correct. This served as our baseline pre-intervention data. The reports were reviewed by two medical students, a PGY-3 radiology resident, and an attending radiologist with fellowship training in abdominal imaging with 11 years of dedicated abdominal imaging experience.

Intervention: creation and implementation of renal mass dictation macros

The abdominal imaging section, in conjunction with our Quality Committee, developed a dictation macro, called “Follow-up renal mass.” Embedded in the macro is a picklist designed to address several possible incidental renal lesions, including simple renal cysts (recommending no additional follow-up), solid renal masses (recommending urology consultation), and a variety of indeterminate renal lesions. The macro was developed using synthesized recommendations from the ACR Incidental Findings Committee and was reviewed with a urology department representative. 

After development and small-group testing of the follow-up macro, we deployed the tool for group-wide utilization. Education materials were disseminated to all radiologists and trainees in the group via email. Materials included a PowerPoint® presentation (Microsoft Corporation, Redmond, USA) detailing a review of current ACR suggestions for the management of incidental renal lesions, as well as how to incorporate the recommendations into radiology reports. Step-by-step instructions were included, describing how to insert the macro and select the appropriate picklist selection during the report dictation, including several illustrative case examples. The materials were distributed via email with one follow-up reminder email two weeks later. Radiologists were encouraged to attest to the review and completion of the materials, which yielded a voluntary attestation rate of 54 out of 95 (56%) of the radiologists in the group. The educational materials were sent to all radiologists, regardless of subspecialty. 

Post-intervention 

Data was collected approximately 12 months after the follow-up macro was released and educational material was disseminated. The same methodology was utilized as the pre-intervention data over a 7-month period, from January 2021 through July 2021. This yielded 501 abdominal MRI or CT reports, 403 of which were excluded by the same exclusion criteria discussed earlier, leaving 98 cases. Again, we identified and assessed the case’s incident report, wherein the lesion was initially detected. The 98 cases were characterized utilizing the same recommendation categories as before: 1) no report recommendation offered, 2) incomplete or discordant recommendation, or 3) correct recommendation. The ACR white paper recommendations for incidental renal lesions were again utilized as the reference standard.

## Results

**Figure 1:**
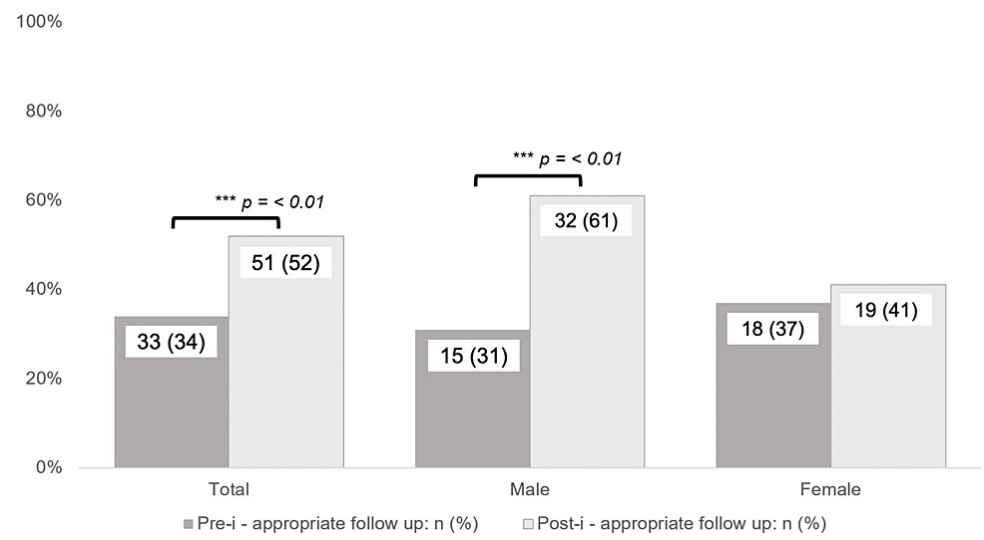
Pre- and post-intervention appropriate follow-up recommendations

Patient demographics and characteristics are listed in Table 2. Pre-macro studies included 49 (50%) males and 49 (50%) females, while the post-macro studies included 52 (53%) male and 46 (47%) female patients with details about follow-up recommendations by sex seen in Figure 1. Age was broken down by decade of life, with 18-30 years and 80+ being their own categories. Patient age ranges for the pre- and post-intervention cohorts are shown described as follows. Pre-implementation studies included three (3%) in 18-30 years, five (5%) in 30-40 years, nine (9%) in 40-50 years, 12 (12%) in 50-60 years, 32 (33%) in 60-70 years, 33 (34%) in 70-80 years, and four (4%) in 80+ years. post-implementation studies included one (1%) in 18-30 years, two (2%) in 30-40 years, 11 (11%) in 40-50 years, 12 (12%) in 50-60 years, 32 (33%) in 60-70 years, 29 (30%) in 70-80 years, and 11 (11%) in 80+ years. 

**Table 2:** Patient demographics

Characteristics	Pre-Intervention (n=98)	Post-Intervention (n=98)	p-value
Age (y), mean ± SD	63 ± 14	65 ± 13	0.24
Sex, male-female ratio	49:49	52:46	0.67
Imaging modality			0.46
CT w/o contrast	13	7	
CT w contrast	47	45	
Ultrasound	33	40	
MRI	5	6	

**Figure 2:**
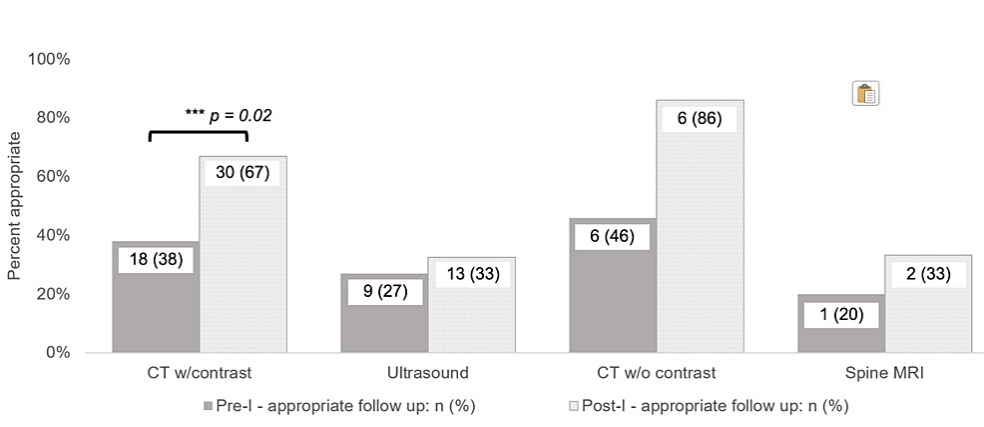
Pre- and post-intervention appropriate follow-up recommendations by imaging modality

The 98 pre-implementation incident exams included CT with contrast 47 (48%), abdominal or renal ultrasound 33 (34%), CT without contrast 13 (13%), and non-contrast or non-abdominal MRI five (5%). The 98 post-implementation incident exams included CT with contrast 45 (46%), ultrasound 40 (41%), CT without contrast seven (7%), and non-contrast or non-abdominal MRI six (6%) (Figure 2). 

Overall, results indicated that pre-implementation studies saw a successful recommendation in 33 (34%) cases, while post-implementation studies showed a successful recommendation in 51 (52%) cases. This demonstrated that implementation of the macro yielded a statistically significant increase in correct recommendations (p = 0.009). Additionally, there was a decrease in the number of incomplete or discordant recommendations from 49 (50%) to 37 (38%) (p = 0.09), and a decrease in the number of reports lacking follow-up recommendations from 16 (16%) to 10 (10%) (p = 0.21) between pre-implementation and post-implementation studies.

## Discussion

Incidental renal masses are a burdensome issue in healthcare. When solid neoplasms are detected incidentally, it offers the potential for intervention at a lower stage, which is beneficial given the correlation between early-stage disease and survivability [10,11]. However, renal lesion detection can also present several management challenges, including inappropriate or poorly timed follow-up, patients being lost to follow-up, and the introduction of costly, wasted steps in more definitive renal lesion characterization and potential treatment. Variability in radiologist reporting of incidental renal lesions presents an additional barrier to providing optimal workups.

The primary goal of this quality improvement project was to assess the reporting of incidentally detected renal lesions before and after deploying a set of standardized dictation macros. Our quality improvement effort yielded a statistically significant improvement in the number of correct follow-up recommendations, increasing from 34% in the pre-intervention cohort, up to 52% following intervention (p < 0.01). Secondarily, we observed a concomitant decrease in the number of cases for which no recommendation was offered. Although group-wide improvement was achieved, there is still significant room for further development. Many diagnoses were made through the use of this standardized process that would have otherwise been missed, most commonly renal cell carcinoma and distant metastasis to the kidneys.

This study also provided insight into radiologists reporting behavior. For example, 32 of the 49 incomplete/discordant recommendations prior to macro implementation were instances wherein the radiologist recommended an MRI upon discovery of a solid renal lesion instead of a urology consultation, making it the most common incomplete/discordant recommendation. When a solid renal neoplasm is discovered on contrast-enhanced CT, it can frequently be fully characterized on that same exam. Thus, while the ACR does not provide specific guidelines for this instance, it logically follows that upon detection of such a lesion, urology consultation is the next best appropriate step. This allows for additional imaging to be ordered by the urologist on an as-needed basis for pre-surgical planning.

**Figure 3:**
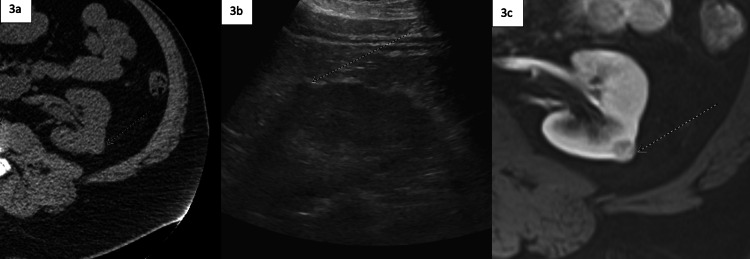
Imaging studies showing workup of indeterminate renal lesion 3a: Unenhanced CT demonstrating an indeterminate density left renal lesions. Note the small size of the lesion and the significant intra-abdominal and subcutaneous adipose tissue. The reading radiologist recommended an ultrasound for characterization. 3b: Renal ultrasound in a poor candidate, upon which the lesion was not able to be definitively characterized as cystic or solid, necessitating the ultimate recommendation of an abdominal MRI. 3c: Contrast-enhanced MRI characterizing the lesion as a small, enhancing malignancy.

Several additional cases were observed wherein the radiologist recommended follow-up ultrasound in an attempt to characterize an indeterminate renal lesion in cases where either the lesion was very small or in patients that were poor ultrasound candidates, as seen in Figure 3a. Very small renal lesions or lesions in patients with a large body habitus, or both, are less likely to be characterized on ultrasound, an example of which can be seen in Figure 3b. If lesion and/or patient selection is inappropriate, a definitive characterizing MRI is likely to be needed. Of note, the follow-up macros do not include an option to recommend an ultrasound, lowering the frequency of extemporaneous ultrasound imaging.

**Figure 4:**
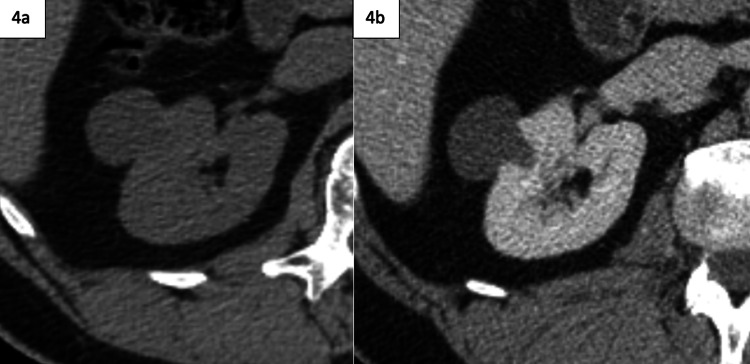
Initial unenhanced CT with follow-up contrast-enhanced CT of renal mass 4a: Unenhanced CT demonstrating a single, exophytic cystic renal lesion measuring less than 20 Hounsfield units, demonstrating the characteristics of a benign renal cyst, requiring no dedicated follow-up. 4b: Follow-up contrast-enhanced CT demonstrating nonenhancement.

Just as there are cases involving renal lesions that require more definitive characterization, there are also cases in which a renal lesion may be appropriately characterized as non-worrisome on initial imaging. In these instances, it is important for the radiologist to avoid ambiguity and unnecessary additional workup, which is displayed in the sequence shown in Figures 4a-4b. Figure 3c in contrast shows an example of a small, enhancing lesion that is clearly confirmed as renal cell carcinoma.

One significant additional point for discussion is where the macros do not precisely match ACR

White Paper Recommendations

There is one instance of this, namely as regards indeterminate, homogenous, non-fat-containing lesions initially discovered on unenhanced CT. The ACR classifies such lesions as either “Indeterminate” or “Indeterminate, too small to characterize” [9]. Their recommendations for indeterminate, too small to characterize lesions include a contrast-enhanced MRI or renal protocol CT, to be completed within 6-12 months. Their recommendation for general indeterminate lesions is a follow-up MRI or CT without a specific timeframe.

Our macros more specifically subclassify these indeterminate lesions and add guidance with regards to size criteria and timing of follow-up. Since the ACR does not directly define what constitutes “too small to characterize” (and therefore merits follow-up within 6-12 months), our group divided small indeterminate lesions into “Indeterminate, too small to characterize” (which we specify as lesions less than 1 cm in diameter) and "indeterminate lesions that measure between 1 and 1.9 cm." For indeterminate lesions measuring less than 1.0 cm, we recommend an MRI or renal protocol CT within 12 months. For indeterminate lesions measuring between 1 and 1.9 cm, we recommend an MRI or CT at 6 months. This additional specificity, both in lesion characterization and in follow-up recommendations is favored to increase clarity and consistency among reports, decrease loss to follow-up, and increase appropriate image ordering. Of note, for indeterminate renal lesions measuring greater than 1.9 cm, we recommend an immediate follow-up MRI or CT, unchanged from the white paper guidelines.

Beyond improving report recommendations, the macros offer several further benefits. Radiologists’ deviation from ACR societal recommendations when encountering incidental findings has been recently evaluated. The most cited reason radiologists deviate is simply that they do not recall the details of the recommendation they are giving [12]. The second, related reason, is a lack of the time required to look up the details for the recommendation [12]. However, our experience demonstrates that for individual subtypes of incidental findings, using a predetermined algorithm coupled with standardized macros for various imaging findings, these challenges can be addressed at the time of initial report dictation.

Additional benefits of the macros include increased clarity and consistency across radiology reports, the frequent inclusion of cited sources, which may increase the clinician’s confidence in the recommendation, and improved detail of recommendations compared to free text dictation. In our experience, many free text recommendations lack either the preferred imaging modality or the timeframe in which follow-up should occur, and in some cases, both. Follow-up macros eliminate this issue by having structured modality and timing built into the recommendation. An additional benefit of macro use is that whenever published societal best practices change, group-wide macros allow for a single source to be updated rather than all radiologists having to implement the updates individually.

Finally, the predictability of the language used in macros allows relatively simple natural language processing programs to track studies in which the macros are used. This facilitates the ability to detect and track important incidental findings, which can be utilized to ensure that patients and referring providers are reminded of upcoming or overdue imaging studies. The ability to track cases puts radiology in a position to contribute to the longitudinal care of patients by ensuring that patients are not lost to follow-up. Blagev et al. showed that even when an appropriate follow-up recommendation was included in the impression for incidentally detected pulmonary nodules at CT angiogram, only 29% of patients returned for follow-up [13]. However, with the aid of natural language processing, robust follow-up programs may assist in ensuring referring providers and patients are aware of recommended imaging follow-up.

Additional, but non-statistically significant, trends were observed in our project. We showed a post-implementation increase in correct recommendations made in free text without the use of the macros. Although not specifically studied, this suggests some of the radiologists may prefer their own language over standardized macros, though the increase in correct recommendations suggests they may have been influenced by the educational material. Finally, we observed the increased appropriateness of follow-up exam timing and a decrease in advanced imaging ordered in the emergency room and inpatient setting. Some of the more common scenarios prior to macro introduction had been the premature workup of a too small to characterize renal lesion, as well as inpatient and/or immediate workup of incidental indeterminate lesions detected in the inpatient or emergency room setting. Each of these scenarios represented inappropriate use of medical resources. Immediate workup of a very small lesion may lead to another indeterminate diagnosis and necessitate an additional longer-term follow-up imaging study. Follow-up in 6-12 months allows the lesion time to potentially grow large enough to allow for definitive characterization, without significant risk of upstaging. It also allows patients to recover from any potential acute illnesses, which might confound an immediate assessment or lead to suboptimal image quality.

**Figure 5:**
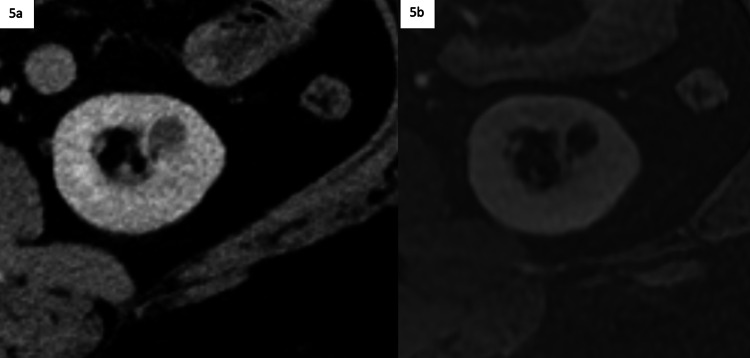
Enhanced abdominal CT with follow-up contrast-enhanced abdominal MRI following admission 5a: Contrast-enhanced abdominal CT ordered in the emergency room for abdominal pain, demonstrating an incidentally detected indeterminate left renal lesion measuring less than 2 cm. 5b: Contrast-enhanced abdominal MRI subtraction image, demonstrating a nonenhancing Bosniak 2 cyst. The MRI was ordered 2 days after the patient was admitted from the ER, during their inpatient stay.

**Figure 6:**
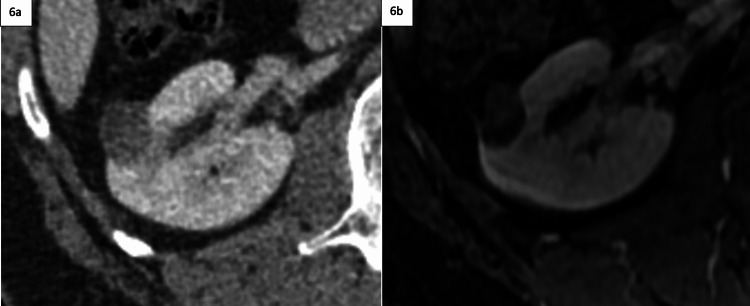
Enhanced CT showing indeterminate lesion with follow-up MRI 8 months later 6a: Contrast-enhanced CT ordered in the emergency room for right lower quadrant pain, demonstrating an incidentally noted indeterminate right renal lesion measuring less than 1.9 cm. The radiologist recommended a follow-up MRI in 6-12 months, utilizing the renal lesion follow-up macros. 6b: Contrast-enhanced abdominal MRI, ordered on an outpatient basis 8 months later demonstrates an enhancing renal malignancy without significant interval growth or invasive features.

Non-emergent follow-up imaging allows for a workup to be completed in a more cost-effective outpatient setting in the instance of lesions being discovered during an emergency room or inpatient admission, which can be seen in Figures 5a-5b and Figures 6a-6b. Potential cost savings were not evaluated in our project but represent an additional opportunity for further investigation.

The study had several limitations. Our patient cohort was created by retrospectively reviewing the initial imaging reports only of patients that underwent dedicated multiphase CT or abdominal MRI exams for the purposes of renal mass characterization. Thus, our study omits patients that never underwent characterizing exams, including patients that were lost to follow-up. The search criteria inherently missed all cases where the use of the correct recommendation appropriately halted workup in both the pre-implementation and post-implementation study groups. For example, a Bosniak 2 cyst, for which a radiologist appropriately recommended no further follow-up, would not have been captured. We also excluded high-risk asymptomatic patients, such as those with tuberous sclerosis or Von Hippel-Lindau syndrome. Additionally, there is a subset of indeterminate renal lesions, namely those thought likely to be cystic in patients that are good ultrasound candidates, that may be successfully characterized on an ultrasound exam. Any such cases, wherein the radiologist did recommend an ultrasound to characterize a lesion, would not be captured in our study population. Finally, the sample size was not adequate to assess some of the secondary radiology report trends that were observed.

Adoption of the macros by radiologists was encouraged, but not mandatory. The educational material was distributed to the entire group, with a single reminder communication, but there was no requirement that the material be reviewed. Fifty-six percent of practicing radiologists attested to completion. We did not account for radiologists for whom this material was unlikely to be relevant (for example, physicians working in 100% breast imaging or interventional radiology that are exposed to few, if any, incidental renal lesions). Additionally, the utilization of the macro is a manual process, so even the most well-intentioned radiologists had to remember to insert and select the best follow-up. We also did not manually review each case to confirm that the appropriate macro had been utilized, trusting the judgment of the initial interpreting radiologist.

## Conclusions

In conclusion, our study demonstrates that the implementation of a standardized set of follow-up macros can significantly improve the quality of follow-up recommendations for incidentally detected renal lesions. The number of reports containing discordant, incomplete, and missing recommendations can also be reduced. This yields higher-quality reports with less ambiguity for referring providers. The incomplete adoption of the follow-up macro system by radiologists may be addressed in the future with machine learning tools that can aid in suggesting appropriate follow-up based on lesion characteristics. Such tools may also lead to improved patient safety, as standardized language improves the ability to track treatment and can more easily be incorporated into patient follow-up programs, reducing the risk of patients with important incidental findings being lost to follow-up.
